# Topochemical engineering of composite hybrid fibers using layered double hydroxides and abietic acid

**DOI:** 10.3762/bjnano.10.60

**Published:** 2019-02-28

**Authors:** Liji Sobhana, Lokesh Kesavan, Jan Gustafsson, Pedro Fardim

**Affiliations:** 1 Laboratory of Fibre and Cellulose Technology, Åbo Akademi University, Porthansgatan 3, FI-20500, Åbo, Finland; 2 Laboratory of Materials Chemistry and Chemical Analysis, Turku University Centre for Materials and Surfaces (MatSurf), University of Turku, Vatselankatu 2, FI-20014 Turku, Finland; 3 Department of Chemical Engineering, KU Leuven, Celestijnenlaan 200F b,us 2424, B-3001 Leuven, Belgium

**Keywords:** abietic acid, composite hybrid fibers, high tensile pulp, hydrophobic pulp, layered double hydroxides

## Abstract

Topochemical engineering of hybrid materials is an efficient way of synthesizing hydrophobic and highly tensile fiber composites by utilizing the intermolecular hydrogen bonds in natural materials. These materials include wood pulp fibers, abietic acid (resin acid) and inexpensive metal salts. In this work, a hybrid composite was created using bleached and unbleached kraft pulp fibers as cellulose platform. In situ co-precipitation of layered double hydroxide (LDH) was performed to grow LDH crystals on the surface of the cellulose fibers, followed by the immobilization of abietic acid (AA) on LDH-grafted cellulose. Here we aimed to benefit from the hydrogen bonding between –OH groups of cellulose and LDH, and the –COOH groups of AA to obtain charge-directed assembly of one material on the other material. Thus, composite hybrid fibers (C-HF) were produced and then characterized by optical (CAM), spectroscopic (XRD, IR) and microscopic techniques (SEM) to determine their average length and distribution, structure and purity, bonding, and morphology. These fibers further were tested for water contact angle (hydrophobicity), oil absorption (lipophilicity), tensile strength and ISO brightness measurements. The performance of C-HF was compared with unmodified reference fibers (REF), fibers composed with only AA (C-F) and LDH-hybridized fibers (HF). The results revealed a variety of correlations between materials and their properties due to characteristic surface morphology, functional groups, hydrogen bonding and natural co-materials such as lignin and hemicelluloses. Attractive and repulsive van der Waals forces between material entities play a crucial role in the resulting properties.

## Introduction

Renewable chemicals or materials and their value addition are the current focus in the area of materials and applied chemistry, which strive to develop new end-products with novel functionalities via chemical bonds and physical interactions [1]. Wood pulp is one of these renewable materials. It is obtained from forest products industries and stands as a source of many polymeric materials such as cellulose, hemicelluloses, and lignin [2]. Cellulose, being one of the most abundant natural polymers, has been a target for basic and applied research [3]. It possesses chemically reactive and physically interactive, primary and secondary –OH groups on its molecular skeleton. This carbon-bonded –OH groups serve as the locations for any sort of modifications [4] in cellulose to produce customized end-use materials. They can be oxidized to –C=O, –CHO and –COOH or reduced to hydrocarbons depending on the aim of the application [5–7]. Physically, these –OH groups make cellulose hydrophilic [8]. In addition, these –OH groups facilitate binding between cellulose fibers strands in wood pulp via intermolecular hydrogen bonding. Thus, they contribute to the moderate inherent tensile strength of the fibers as well [9]. On the contrary, there has been always a research motivation to make cellulose hydrophobic [10–12] with high tensile strength [13].

Topochemical engineering is a method of designing the fractionation (disassembly) and fabrication (assembly) of highly engineered functional materials using a combination of molecular and supramolecular techniques. Topochemical engineering is inspired by bioassembly observed in natural systems such as trees and microorganisms [14]. The present work utilizes the approach of hydrogen bonding with external hydrophobic moieties in order to bring water-repelling properties to the cellulose fiber surface. In this work, abietic acid, a hydrophobic renewable material was combined with cellulose via a structure-directing agent, i.e., a layered double hydroxide (LDH). LDHs, inorganic ceramic materials carry symmetrically distributed charge centers/hydrogen bonds. This idea was the extrapolation of our earlier reported work, where introducing hydrophobicity was carried out by anchoring a hydrophobic moiety, namely stearic acid (SA), on wood pulp via a bridging LDH molecule. This led a new hybrid organic–inorganic–organic composite (SA-LDH-CEL) [15].

There are two different methods to make cellulose hydrophobic: (1) chemical methods such as esterification or etherification, and (2) physical methods such as composite development using hydrophobic moieties of silicones, fluorocarbons, or surfactants. The physical methods employed to make cellulose hydrophobic were applied only to regenerated cellulose and not to pristine wet-state cellulose. Our earlier reported work directly utilized the wet pulp fibers without any prior treatment. Moreover, anionic surfactants cause environmental pollution when the resultant acidic materials are washed out. Therefore, the choice of stearic acid was motivated by green-chemistry principles. This method differed from previous reported methods where hydrophobic molecules were directly incorporated into pulp fibers, insofar as it utilized the charge centers of LDH to make a conjugation with stearic acid on one side of the LDH molecules and cellulose on the other side. This way the amount of adhesion of hydrophobic molecules on pulp was carefully controlled as the adhesion was steered by self-directed –COOH^…^OH hydrogen bonding. This SA-LDH-CEL hybrid material offered super-hydrophobicity.

We have decided to extend this research using another environmentally friendly natural fatty acid, namely abietic acid (AA). Abietic acid is a resin acid, abundantly present in pinewood and other coniferous plants [16]. Rosin, the colorful resin material used as pigments in inks, varnishes and adhesives, largely consists of abietic acid. It is highly hydrophobic and soluble only in organic solvents such as acetone, ethanol and diethyl ether. The reason for choosing AA was that it contains two more carbon atoms (C_20_H_30_O_2_) in its skeleton (19 C-chain) with cyclic structure and unsaturated π-bonds. Moreover, compared to stearic acid, abietic acid is less flammable, insoluble in water and denser than water, and has a high melting temperature. These properties can yield improved hydrophobicity and mechanical strength to the fibers.

Layered double hydroxides are hydroxylated mixed metal salts (clay minerals) that have unique fascinating three-dimensional structures in which positively and negatively charged ions are stacked in a uniform fashion [17–19]. This makes the material employable for charge-directed self-assembly of any molecule of interest on its surface. The metals in this kind of inorganic solids, such as Ni, Mg, Zn, Al, Fe, usually have oxidation states of +2 and +3. The anions in these materials are commonly CO_3_^2−^, Cl^−^, NO_3_^−^. These interlayer anionic sites are highly tunable, thus, they can be optimally replaced with anions of our interest [20–25]. The hydroxyl groups present on the brucite layers of LDHs are able to form hydrogen bonding with foreign molecules to increase stability. The present work focuses on stacking abietic acid and cellulose on each side of LDHs via van der Waals forces of hydrogen bonding. Thus, LDH works as an assembling agent.

The raw materials used in this study were bleached pine kraft pulp and unbleached spruce kraft pulp. Bleaching is a process in which pulp fibers are treated with strong oxidative chemicals such as peroxides or perchloric acid. This process removes most of the lignin and some hemicelluloses from the pulp, making it soft and flexible. These co-materials are detrimental to functionalizing cellulose and its utilization as technical material. Both bleached and unbleached pulps further undergo refining. Refining is a process in which mechanical compression and shear forces are applied to the intact wet fiber network in order to increase the surface exposure and surface area. In addition, it opens up fibrils on the surface, which increase the surface roughness of the fibers and yield good adhesion properties. Bleached and unbleached fibers, refined and unrefined, were used as starting materials to synthesize direct composites (AA + fiber), hybrid fibers (LDH + fiber) and composite hybrid fibers (AA + LDH + fiber). We expect that these materials show different properties with regard to water repellency, oil absorption, tensile strength, and optical brightness. Composing renewable materials such as cellulose and abietic acid for customized product development is in the line of interests of circular-economy initiatives implemented by governments and research funding agencies all over the world.

The originality of this study is that it uses chemically (bleached)/mechanically (mill-refined) treated fibers in their pristine wet state. They are pine (bleached) and spruce (unbleached) kraft pulp fibers, in contrast to our previous study in which birch (unbleached) kraft pulp was used. The commercially acquired pulp fibers were first disintegrated, and further refined mechanically prior to LDH deposition. During LDH preparation, sodium carbonate was used as CO_3_^2−^ source instead of urea. The AA-modified LDH-F hybrid material preparation method was slightly modified due to solubility difference between AA and SA. Also, in our earlier work, stearic acid treatment on HF was carried out for 24 h, whereas this work needs only 15 min to modify the surface of HF. Thus, the time spent on material development was drastically reduced. In the present work, the degree of refinement of fibers, fiber length distribution/average fiber length evidenced from optical microscopy and the optical brightness of the fibers were measured in a first attempt.

## Experimental

### Materials

Bleached and unbleached kraft pulps (wood fibers) from pine and spruce, respectively, were used as the platform materials for inducing hydrophobic behavior and high tensile strength. Both unrefined and refined pulps were investigated in the study. Abietic acid (C_19_H_29_COOH, Fluka) and sodium hydroxide (NaOH, 97%, VWR) were purchased. Metal nitrate salts (magnesium nitrate hexahydrate Mg(NO_3_)_2_·6H_2_O, aluminium nitrate nonahydrate Al(NO_3_)_3_·9H_2_O, ethanol C_2_H_5_OH) and sodium carbonate were procured from Sigma-Aldrich. All these chemicals were used as received without any further purification.

### Methods

The present work employed some protocols that were already tried out in our earlier reported work. Those methods are 1) pulp disintegration, 2) LDH preparation in the presence of pulp fibers, 3) water contact angle measurements, 4) oil absorption measurements, 5) tensile strength measurements, 6) fiber handsheet making. The remainder of the protocols are reported here for the first time.

#### Wet disintegration of cellulose pulp

30 g of dry pulp (bleached/unbleached) was soaked in 2 L of water for 4 h followed by loading into a disintegrator with 30,000 revolutions (ISO 5263-1:2004) to break the intertwined cellulose fiber networks and disassemble the fibers from each other. The disintegrated pulp was dewatered by centrifugation to yield the free unbound cellulose fibers. These cellulose fibers were used for further grafting with LDH.

#### Refining of the pulps and Schopper–Riegler analysis

Refining of bleached and unbleached pulps was performed at a pulp concentration of 1.57% (w/w) according to ISO 5264-1:2012 using a Valley laboratory beater. The bleached pine and unbleached spruce pulps were refined for 60 min and 90 min, respectively, after which the pulps were collected for further analyses and treatment. In Table 1 the abbreviations used for the pulp fibers throughout the report are given.

**Table 1:** The following abbreviations will be used to distinguish the different pulps.

Abbreviation	Pulp sample

BKP	bleached kraft pulp of pine, unrefined
BKPR	bleached kraft pulp of pine, refined for 60 min
UBKP	unbleached kraft pulp of spruce, unrefined
UBKPR	unbleached kraft pulp of spruce, refined for 90 min

The water drainability of the refined pulps was determined according to ISO 5267-1:1999, the Schopper–Riegler method, in order to verify the refining degree of the pulps. The refining results in compression, fibrillation and finally intertwinement of the fibrils. Flattened well packed and stacked fibers that decrease the free path of water during filtration are obtained. Thus, refining increases water retention of the wet fiber mat during the Schopper–Riegler measurement, expressed as a higher value of the refining degree in °SR.

#### Grafting of LDH on cellulose fibers

The unbound cellulose fibers as prepared above were hybridized with Mg–Al LDH through in situ co-precipitation. Typically, 3.0 g of disintegrated fibers (oven dry mass) was dispersed in a flask containing mixed metal-salt solutions (Mg and Al nitrate, molar ratio 3:1) under gentle stirring until a homogenous slurry was obtained. Aqueous solution of sodium carbonate was added dropwise to the reaction flask under constant magnetic stirring to ensure homogeneity of the reaction medium. The reaction was performed at room temperature and air atmosphere. The pH value of the co-precipitation medium was kept constant (pH 8.3 ± 0.3) by the simultaneous addition of 2 M NaOH. Then, the resultant slurry was aged in a digestion bomb at 120 °C for 24 h for the crystallization of LDH on cellulose fibers. Thus, Mg–Al LDH was synthesized in the presence of cellulose fibers and subsequently grafted on these fibers uniformly. The LDH-grafted cellulose fibers were filtered and washed repeatedly to remove any unbound LDH particles until a neutral pH value was attained. The recovered wet fibers were conjugated with Mg–Al CO_3_ LDH. (Two more experiments were conducted with varying ratios between metal salts and sodium carbonate). All the experiments were repeated to confirm the reproducibility of the results.

#### Making of highly tensile hybrid fiber handsheets

The LDH–cellulose (LDH-F) inorganic–organic hybrid fibers were made into handsheets of hybrid fibers (HF) of 120.0 gm^−2^ grammage as follows: The hybrid fibers suspension was diluted to a total volume required to get a grammage of 120.0 g·m^−2^. Aliquots of 2 L of suspension were filled in a handsheet maker equipped with a 125 mesh size wire cloth. Water circulation was not applied in the handsheet maker as the fibers were already dispersed uniformly in the pulp solution. The newly formed wet hybrid fiber handsheets were pressed twice at a force of 400 ± 10 kPa between couch blotters for increased drying. Further, these handsheets were conditioned at 23 °C temperature in a room with 50% of relative humidity for 2 days.

#### Functionalization of LDH grafted cellulose fiber handsheets by abietic acid (AA)

On the surface of the 120.0 g·m^−2^ grammage hybrid fiber handsheets (HF) abietic acid (AA) was immobilized to induce hydrophobicity and increased tensile strength. The handsheets were immersed in a tray containing an ethanolic solution of abietic acid (0.1 M) for 15 min under ambient conditions to allow for the grafting between LDH and AA. Then the handsheets were gently taken out and allowed to dry at room temperature.

#### Characterization

**Optical microscopy analysis:** An optical microscope Nikon Eclipse E200 combined with a Nikon DS-Fi2 digital camera was used to visually analyze the fibrillation level of the fibers.

**Fiber length distribution:** The average fiber length and the length distribution were determined in diluted aqueous solution by using a Kajaani Fiber Lab fiber analyzer (Valmet Automation). The fiber length measurements were performed in duplicates. The contour length of the fibers (LC), i.e., the real length of a fiber, regardless of shape and the length weighted average fiber length, *L*(*l*), were reported.

**X-ray diffraction:** Powder X-ray diffraction (PXRD) patterns of the fiber samples were recorded with a Siemens D501 diffractometer with Cu Kα radiation (λ = 0.15415 nm). Patterns were recorded in the 2θ range of 5–70° in steps of 0.04° with a counting time per step of 8 s. The modified fibers were analyzed by pressing them gently onto a copper sample holder.

**ATR-FTIR analysis:** Attenuated total reflectance Fourier transform infrared (ATR-FTIR) spectra were recorded for samples in the range of 400–4000 cm^−1^ in a Thermo Scientific Nicolet iS50 instrument (Thermo Scientific, Madison, WI, USA) equipped with a diamond crystal and a pressure gauge. A total of 64 scans was recorded and corrected by the Omnic spectral suite software that provided ambient background and ATR correction for 45° incidence angle and one refraction assuming a refractive index of 1.50 for all samples.

**SEM analysis:** A Leo Gemini 1530 field-emission scanning electron microscope with in-Lens detector (LEO Electron Microscopy, Oberkochen, Germany) was used for the characterization of fiber surfaces. The samples were coated with carbon in a Temcarb TB500 sputter coater (Emscope Laboratories, Ashford, UK), the optimum accelerating voltage was 2.70 kV.

### Material testing

#### Water contact angle measurements

The hydrophobicity of reference and modified cellulose handsheets was assessed by contact angle measurements (CA). These were performed with an optical Contact Angle Meter, CAM 200 (KSV Instruments Ltd, Finland), using deionized water. A water droplet volume of 1.6 ± 0.2 μL was placed on a 1 × 10 cm^2^ sample strip. Then the CA values were collected at 200 ms intervals in the beginning and subsequently at 1 s and 2 s intervals until either the water droplet was absorbed or no changes during wetting occurred. The results were analyzed and interpreted with Attention Theta software (Biolin Scientific, Sweden) based on the Young-Laplace function for iterative CA calculation.

#### Oil absorption capacity measurements

To investigate the hydrophobicity of the modified cellulose fibers, pine oil was selected for sorption experiments. The sorption experiments were carried out at room temperature as follows: Small pieces of the pre-weighed cellulose handsheet samples were dipped in oil (10 mL) for approximately 15 min, and then hung in the air for 5 min to let the surface residual oil drip off before being weighed. The oil removal was optimized in such a way that after 5 min, there was no oil dripping from the sheet as well as no surface wetness of stagnant oil observed. Hence, the weight increase was purely by the immobilized oil on the sheets due to hydrophobicity. The increment in oil absorption was calculated as percentage value [(weight after oil absorption − weight before oil absorption)/weight before oil absorption] × 100.

#### Tensile strength measurements

The tensile strength measurements were conducted by using a L&W tensile tester SE 060 according to ISO 1924-2:2008, with a slight variation of elongation rate (12 mm·min^−1^) and test span (100 mm). The tensile tests were performed at 23 °C and 50% RH. Tensile strength tests were also performed with an unmodified ISO standard (ISO 527-2 1BA) sample sheet for comparison. The tensile strength index, calculated as tensile strength divided by grammage, was defined as the inherent strength of the fiber network.

#### Optical brightness measurements

An Elrepho spectrophotometer (Elrepho SE070R, Lorentzen & Wettre) was used to determine the ISO brightness value of the dry fiber materials made into sheets. Both reference and modified fiber sheets were analyzed. The ISO brightness tests were performed at 23 °C and 50% RH.

## Results and Discussion

Bleached (pine) and unbleached (spruce) kraft pulp fibers (F), refined (BKPR, UBKPR) and unrefined (BKP, UBKP), were modified to induce hydrophobicity and higher tensile strength by linking abietic acid (AA) via layered double hydroxide (LDH). The straightforward method to synthesize this composite hybrid fiber (CHF) would be to graft LDH on the fibers first by co-precipitation and to add AA in the same the solution environment under hydrothermal conditions afterwards. However, abietic acid is insoluble in the pre-formed LDH-F solution, and also lacks the capability to spread evenly to form a homogeneous handsheet. Hence, the LDH-F hybrid fibers were first pressed into handsheets (HF) and then modified by immersing it in ethanolic solution of AA. This way, randomly formed clots of AA on the surface of the HF sheets were avoided and the distribution of AA on the fiber surface was improved. Thus, composite hybrid fiber (C-HF) handsheet with homogenous surface and uniform appearance was obtained. Table 2 shows names of series of pulp fibers (control samples and final products) studied, with their abbreviations used throughout this section.

**Table 2:** Samples studied in the present investigation. Note: Bleaching of the fibers was not done at the laboratory. They were obtained as such commercially. Whereas refining was done at the laboratory.

Sample	Pine		Spruce	
	BKP	BKPR	UBKP	UBKPR

reference fibers (unmodified fiber)	REF	REF	REF	REF
composite fibers (AA on fiber)	C-F	C-F	C-F	C-F
hybrid fibers (LDH on fiber)	HF	HF	HF	HF
composite hybrid fibers AA on (LDH-Fiber)	C-HF (0.15 M)	C-HF (0.15 M)	C-HF (0.15 M)	C-HF (0.15 M)
	C-HF (0.30 M)	C-HF (0.30 M)	C-HF (0.30 M)	C-HF (0.30 M)
	C-HF (0.45 M)	C-HF (0.45 M)	C-HF (0.45 M)	C-HF (0.45 M)

### Macroscopic properties

#### Degree of refining of fibers

Schopper–Riegler (SR) analysis showed that refining the bleached kraft pulp of pine (BKPR) resulted in a slightly higher refining degree (higher Schopper–Riegler value) than refining the unbleached Kraft pulp of spruce (UBKPR). BKPR had a SR value of 26 °SR after 60 min refining, whereas the corresponding value for UBKPR pulp (after 90 min of refining) was 21 °SR. The ratio between the degree of refining values of BKPR and UBKPR was 1.24:1. Thus, the increase of refining degree due to bleaching was 24%. The refining degrees of both pulps were considered to be moderate. The refining time is of lesser importance than the refining degree (°SR). Spruce UBKP is a more durable material towards refining than pine BKP. Hence, spruce UBKP needs a longer refining time to reach the same treatment level as pine BKP. Consequently, similar refining degrees (26 °SR and 21 °SR for BKPR and UBKPR, respectively), were achieved. This enables us to exclude refining as an additional parameter to further analysis of the subsequent treatments of the refined fibers.

#### Fiber length

**Figure 1:**
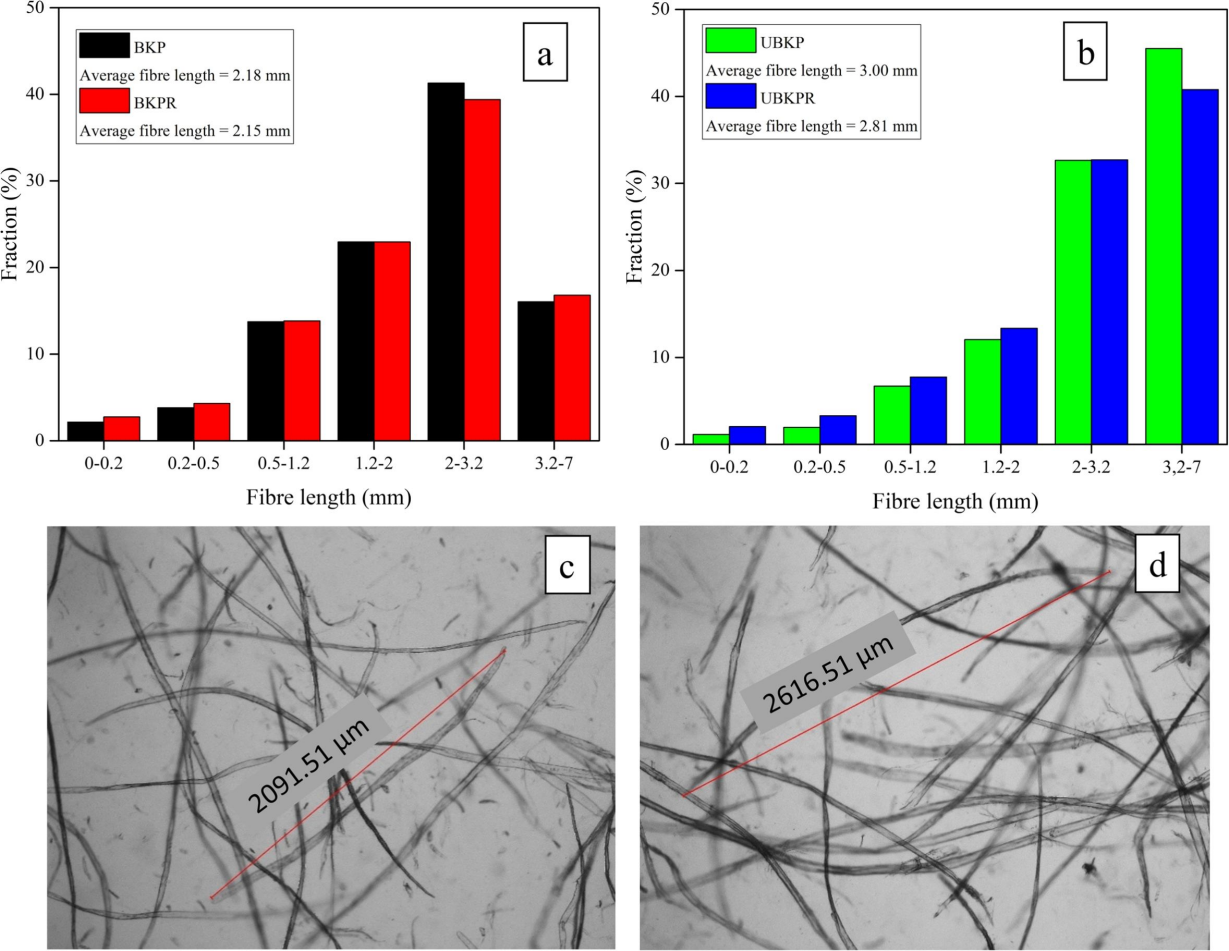
Fiber length distribution of (a) BKP and BKPR and (b) UBKP and UBKPR. Optical microscopy images (10× magnification) of (c) BKPR after 60 min of refining and (d) UBKPR after 90 min of refining.

BKP, UBKP showed average fiber lengths of 2.18 mm and 3.00 mm, respectively (Figure 1a,b). As expected, the average fiber lengths *L*(*l*) of BKPR and UBKPR were smaller (2.15 mm and 2.81 mm, respectively) than those of BKP and UBKP, due to the refinement. The magnitude of the refining effect was larger for unbleached fibers (Δ*L*_UBKP_(*l*) = 0.19) than for bleached fibers Δ*L*_BKP_(*l*) = 0.03). This might be because bleached fibers were already softened and shortened during the chemical treatment.

The fiber length (LC) distribution and average fiber length *L*(*l*) revealed that refining has shortened the average fiber length to some extent for both pulps BKPR and UBKPR. Both refined pulps showed a slight increase in the count of shorter fibers. The increase of these short fibers (0–0.5 mm) was from 3% to 5% for UBKPR (Figure 1b) and from 6% to 7 % for BKPR.

Optical microscopy pictures (10× magnification) showed that external fibrillation had occurred in both refined pulps, BKPR and UBKPR (Figure 1c,d). External fibrillation is the phenomenon in which fibrils stand out from the fiber surface but are still attached to the fiber strands. External fibrillation increases the specific surface area of the fibers and the number of sites for functionalization/grafting/adsorption. In addition, external fibrillation increases inter-fiber bonding which might lead to an increase in tensile strength of the fiber networks. Small separate pieces beaten out from fiber strands are also visible in the microscopy images of both the refined pulps, verifying the presence of the fine fractions (0–0.5 mm), measured in the fiber length investigations.

### Spectroscopic studies

#### Structure and purity

**Figure 2:**
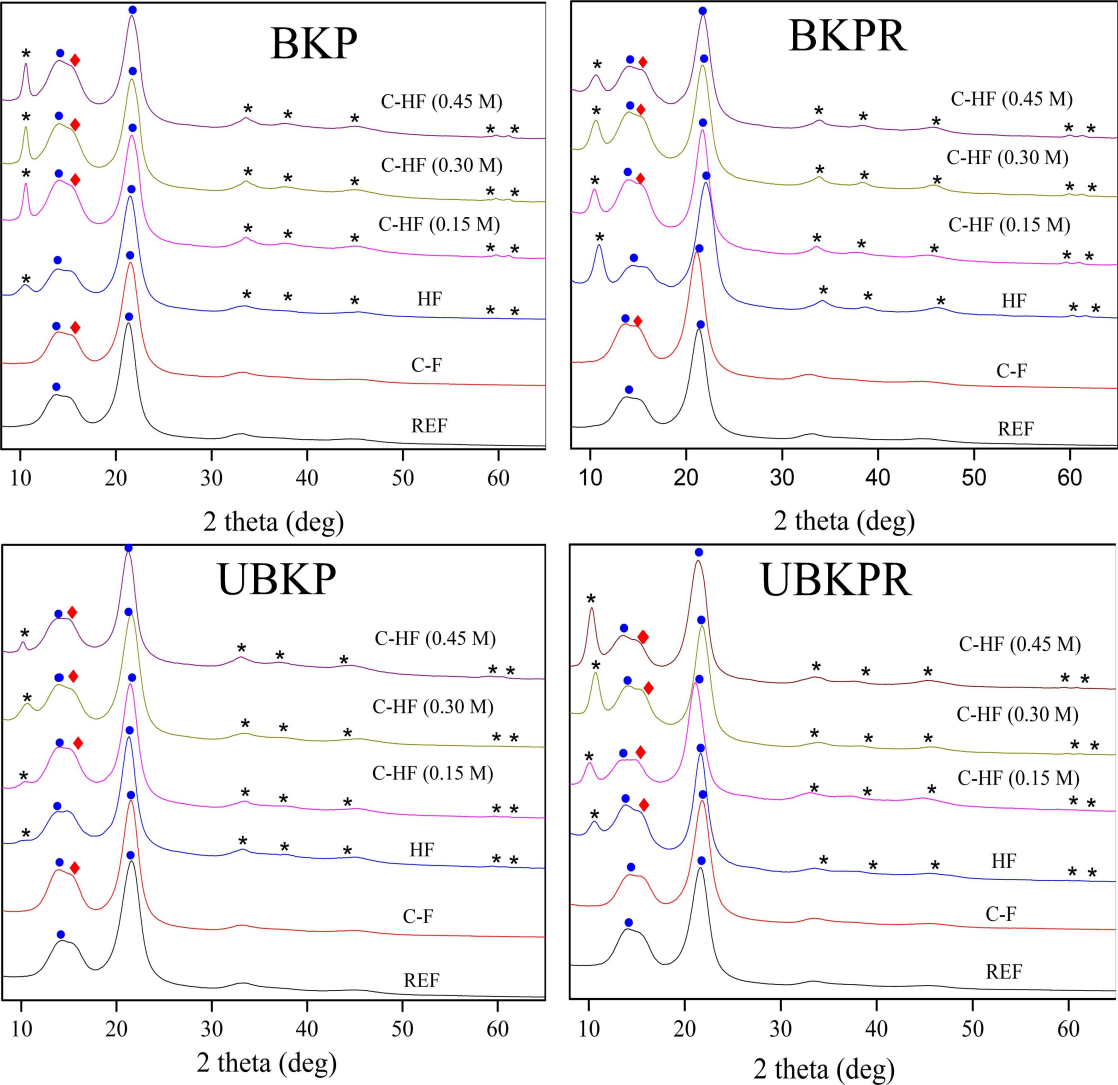
XRD spectra of pulp fibers (filled circles: fibers, asterisks: LDH, filled diamonds: abietic acid).

X-ray diffraction patterns of the pulp fibers (REF, C-F, HF, C-HF_0.15M_, C-HF_0.30M_, and C-HF_0.45M_) are shown in Figure 2.

The C-F and C-HF fibers exhibit characteristic peaks of AA at 15.51°. The presence of LDH in HF and C-HF was confirmed by its characteristic diffractions corresponding to the (003), (006), (012), (015), (018) planes [26]. These diffractions reveal the formation of a crystalline layer structure in the hybrid fibers (HF). The diffraction peaks at 14.5°, 16.5°, and 21.90° correspond to the (110) and (200) crystallographic planes of crystalline cellulose fibers (F) [27–28]. Thus, the presence of AA, LDH, and cellulose in C-F, HF, and C-HF materials, was confirmed by XRD. Out of these three components, AA and cellulose (pulp) were commercially obtained ingredients, whereas LDH was synthesized in situ. XRD might be helpful for gathering more information about formation, purity and grafting on cellulose of LDH.

The characteristic *d*_003_ line spacing of 0.77 nm in LDH disproves any undesirable anion exchange or chelation in the interlayer galleries of LDH. Layered double hydroxides are highly prone to ion displacement reactions at its interlayer anionic sites (e.g., NO_3_^−^, CO_3_^2−^) when it is synthesized in the presence of foreign molecules such as, in this case, cellulose. In this present work, cellulose existed in the neutral form and not as anion. In addition, abietic acid, which could form anions in solution, was only introduced after the hybrid fibers were transformed into sheets. Hence, abietate anions could not replace CO_3_^2−^ in the LDH interlayers, causing contamination or reactions with cellulosic –OH groups.

AA was only connected to the HF fibers through freely available brucite layers (cations on the top plane) of LDH through hydrogen bonding between –OH groups of LDH and –COOH groups of AA. The –OH groups present in the bottom plane of LDH brucite layers were already utilized to graft cellulose through cellulosic O–H polarization. Thus, LDH act as a perfect structure-directing agent in C-HF materials. The XRD spectra of C-HF show a convergence of diffraction peaks of cellulose and AA at 14–16°, a shift from 14.77° to 15.20° and enhanced intensity, which confirm the AA-modification of HF.

#### Structure and bonding

**Figure 3:**
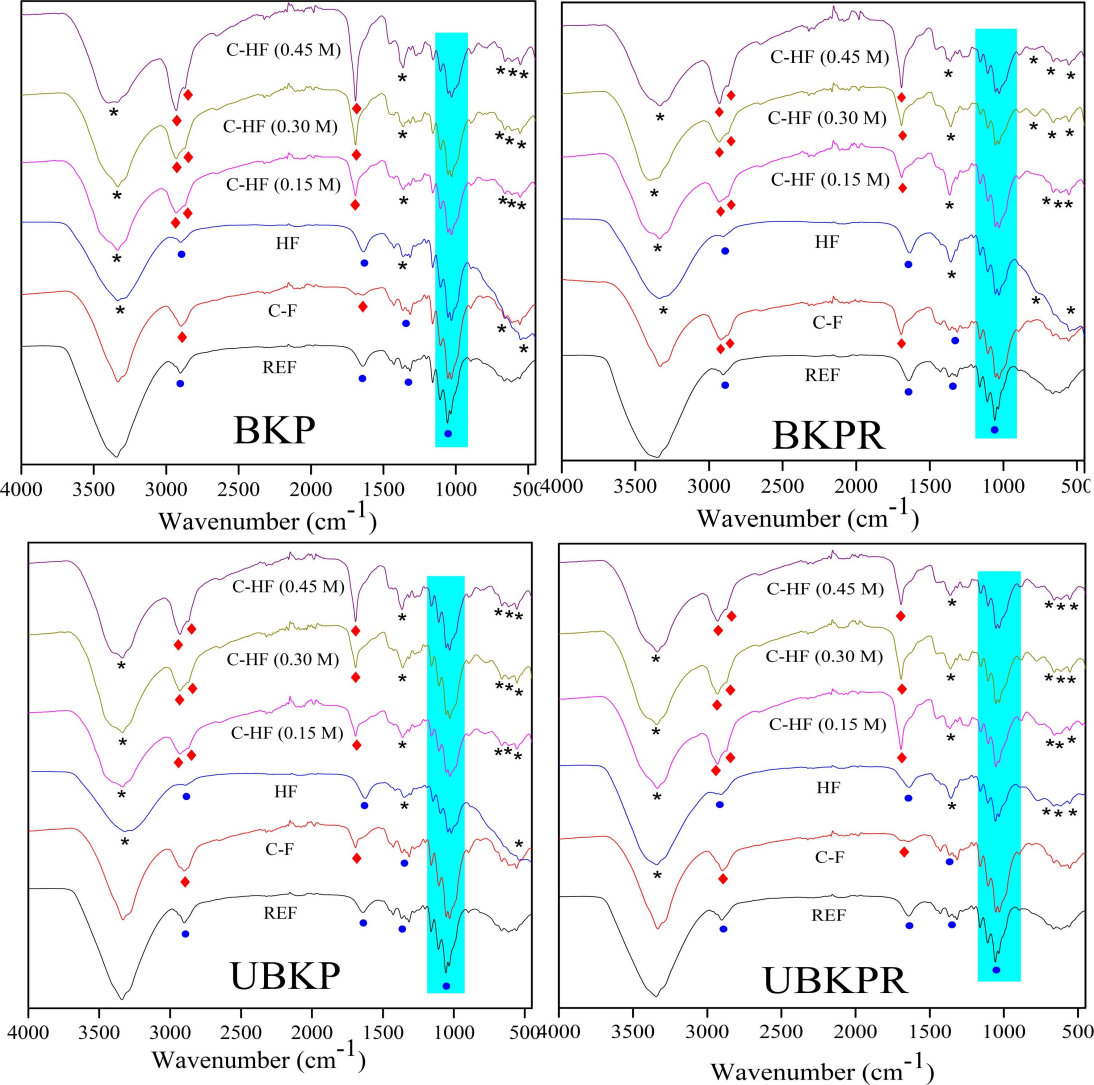
FTIR spectra of pulp fibers (filled circles: fibers, asterisks: LDH, filled diamonds: abietic acid).

The infrared absorption measurements were studied for the control samples (REF, C-F, HF) and the final material, hybrid fiber composites (C-HF_0.15M_, C-HF_0.30M_, and C-HF_0.45M_, Figure 3. The control samples and composite hybrid fibers show their characteristic peaks at 3500–3000 cm^−1^ and 1640 cm^−1^, which are due to stretching and bending of –OH bonds in cellulosic –OH groups/intramolecular hydrogen bonds and in adsorbed water, respectively. The peaks at 897 cm^−1^, 1117 cm^−1^ and 1163 cm^−1^ were assigned to the C–O–C stretching in β-glycosidic bonds, C–C stretching and the C–O–C glycosidic ether band of cellulose, respectively. The cyan portion in Figure 3 denotes the cellulose fingerprint region.

HF and C-HF show a new vibration frequency at 1362 cm^−1^ that corresponds to asymmetric stretching of carbonate anions in LDH interlayer galleries. The frequencies at 617 cm^−1^, 656 cm^−1^ and 783 cm^−1^ were attributed to M–O–M, M–OH, and O–M–O bond vibrations of LDH. The characteristic peaks of AA in C-HF are those at 2936 cm^−1^, 2650 cm^−1^, 1695 cm^−1^, 1670 cm^−1^ and 1277 cm^−1^. The vibration at 2936 cm^−1^ corresponds to C–H stretching absorption bands of –CH_3_, –CH_2_–CH and =CH. The band at 2650 cm^−1^ is due to intermolecular hydrogen bonding in –OH of dimeric –COOH groups. The band at 1695 cm^−1^ is the outcome of C=O stretching vibrations of acid groups, whereas 1670 cm^−1^ is due to C=C conjugated bonds and 1277 cm^−1^ is from C–O deformation vibrations of –COOH. Consequently, the presence of characteristic vibrational frequencies of functional groups confirmed the presence of cellulose, LDH and AA in the respective materials.

### Microscopic Studies

#### Morphology

Scanning electron microscopy (SEM) observations of the pulp fibers (REF, C-F, HF, C-HF_0.15M_, C-HF_0.30M_, and C-HF_0.45M_) revealed how LDH is anchored on the fiber surface and the resin acid AA modifies the surface of hybrid fibers (HF).

**Figure 4:**
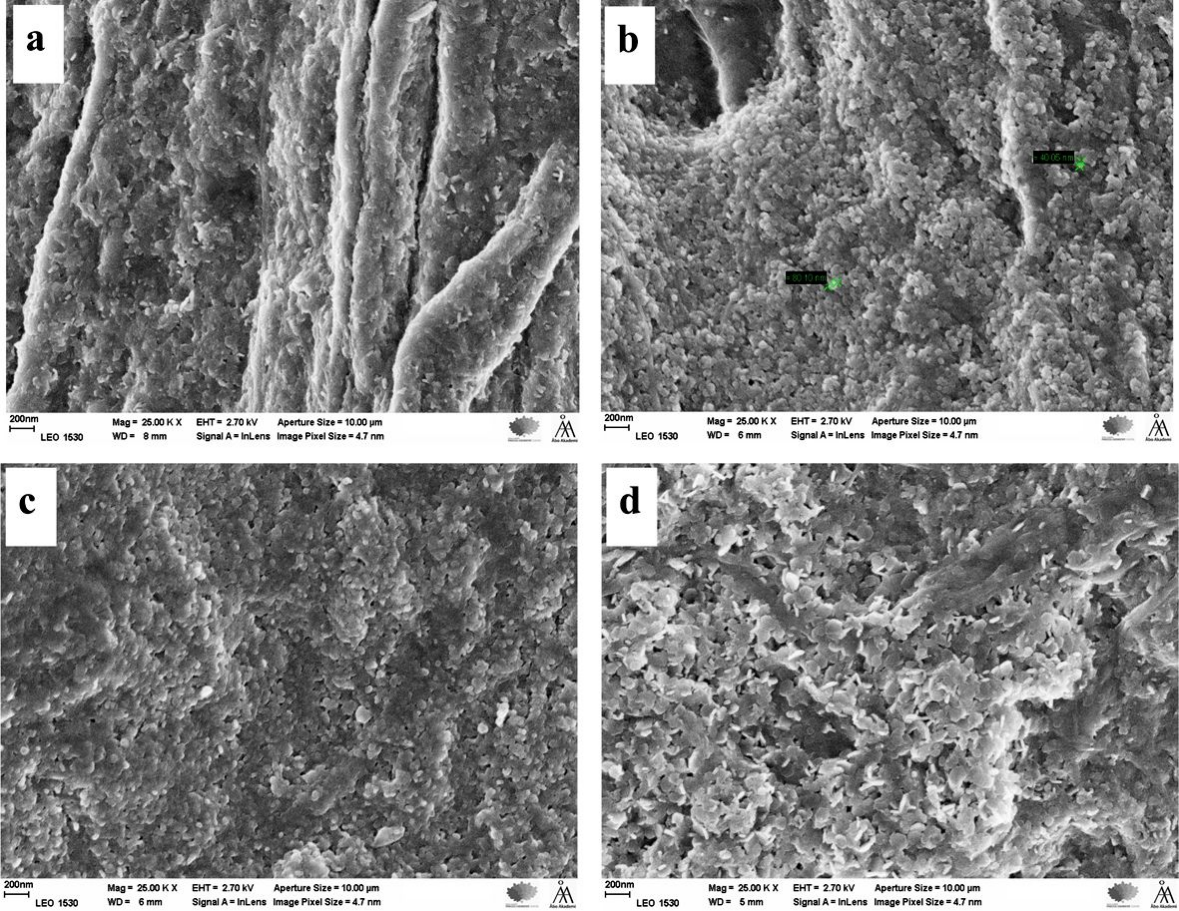
SEM images showing the hybridization of LDH particles on the fiber surfaces of (a) BKP, (b) BKPR, (c) UBKP and (d) UBKPR.

**Hybrid fibers (HF):** The surface characterization of co-precipitated LDH on pulp fibers (HF of BKP, BKPR, UBKP, and UBKPR) revealed that LDH crystals had grown extensively on the fiber surface covering the fibers completely (Figure 4). The HF of bleached fibers (BKP, BKPR) show distinct fiber strand morphologies with LDH particles deposited like a tight continuous solid around the fibers (Figure 4a,b). Whereas HF of unbleached fibers (UBKP, UBKPR) do not show any fiber strands but only solid LDH grains (Figure 4c,d). The HF of refined fibers (BKPR, UBKPR) show LDH crystals with clear shapes like sand pebbles and hexagonal platelets on their surface. The refined fibers have numerous fibrils protruding from the pulp fiber strand. LDH grows around these fibrils and the inter-fibril spaces remain empty. Hence, the LDH crystals are slightly far apart and present in distinct shapes on the refined fibers. Thus, pure hybrid fibers (HF) obtained from direct co-precipitation of LDH on pulp fibers were confirmed by SEM.

**Figure 5:**
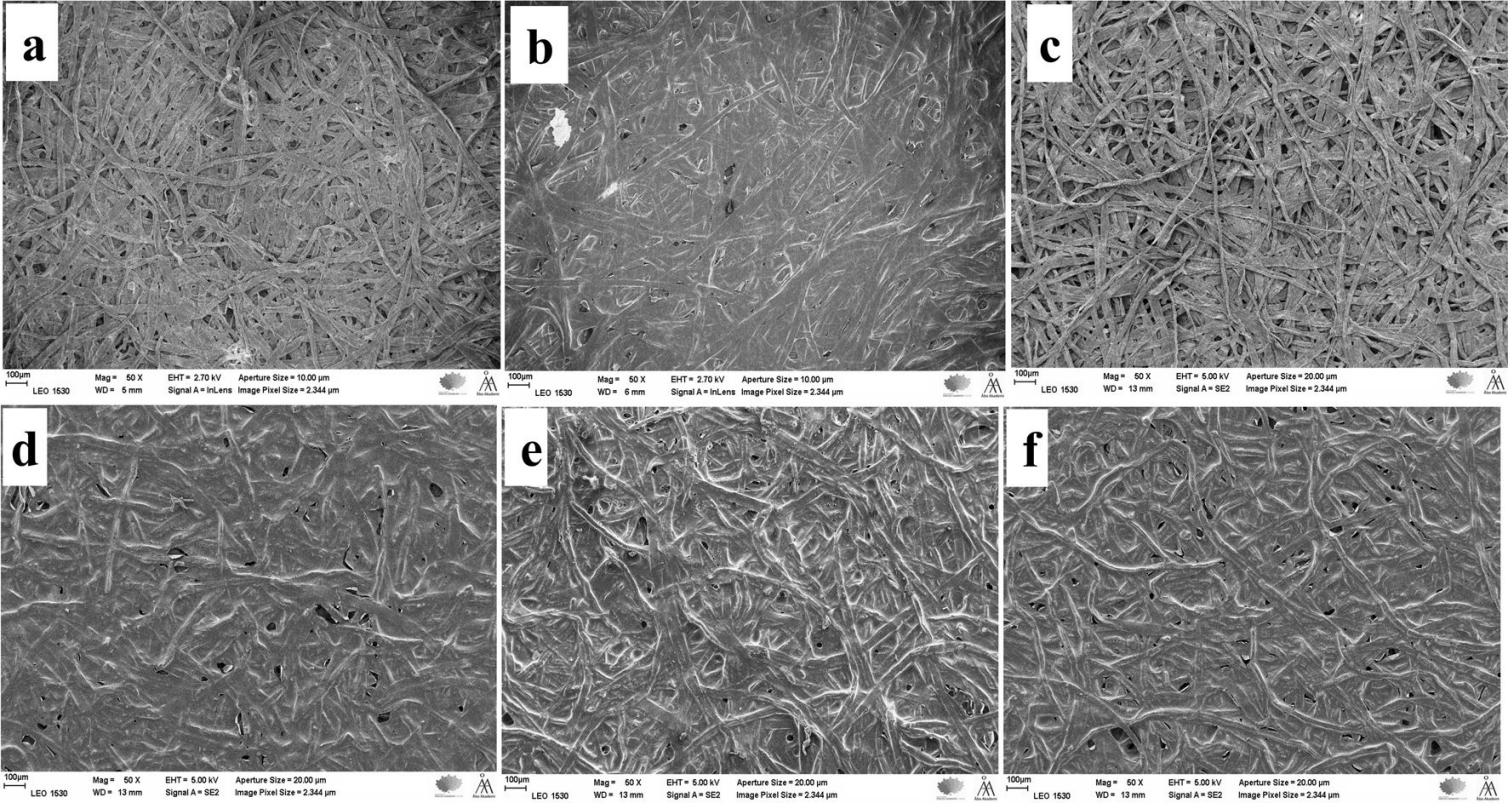
SEM images of the BKP fibers: (a) REF (b) C-F (c) HF (d) C-HF (0.15 M) (e) C-HF (0.30 M) (f) C-HF (0.45 M).

**BKP:** The low-magnification images (Figure 5) of the reference (REF) show a dense, complex web of thin and long cellulose fibers. The surface of REF fibers directly modified by AA (C-F) looks like wax-smeared gauze. Although AA was coated directly on the fibers, it still reveals the complex web of fibers (Figure 5b). The REF fibers hybridized by LDH (HF) (Figure 5c) look very similar to unmodified reference fibers (REF). Figure 5d–f are images of AA-modified HFs, i.e., composite hybrid fibers (C-HF) with varying concentrations of LDH in increasing order (C-HF_0.15M_, C-HF_0.30M_, and C-HF_0.45M_). All these images clearly portray the complete coverage of AA on hybrid fibers (C-HF), again like wax-smeared gauze. The high-magnification images (Figure S1, Supporting Information File 1) display a wrinkled surface of uncoated fibers (REF, HF, Figure S1a,c in Supporting Information File 1) and a very thick, smooth, and shiny layer of AA coating on C-F and C-HF fibers (Figure S1b and Figure S1d–f, Supporting Information File 1).

**Figure 6:**
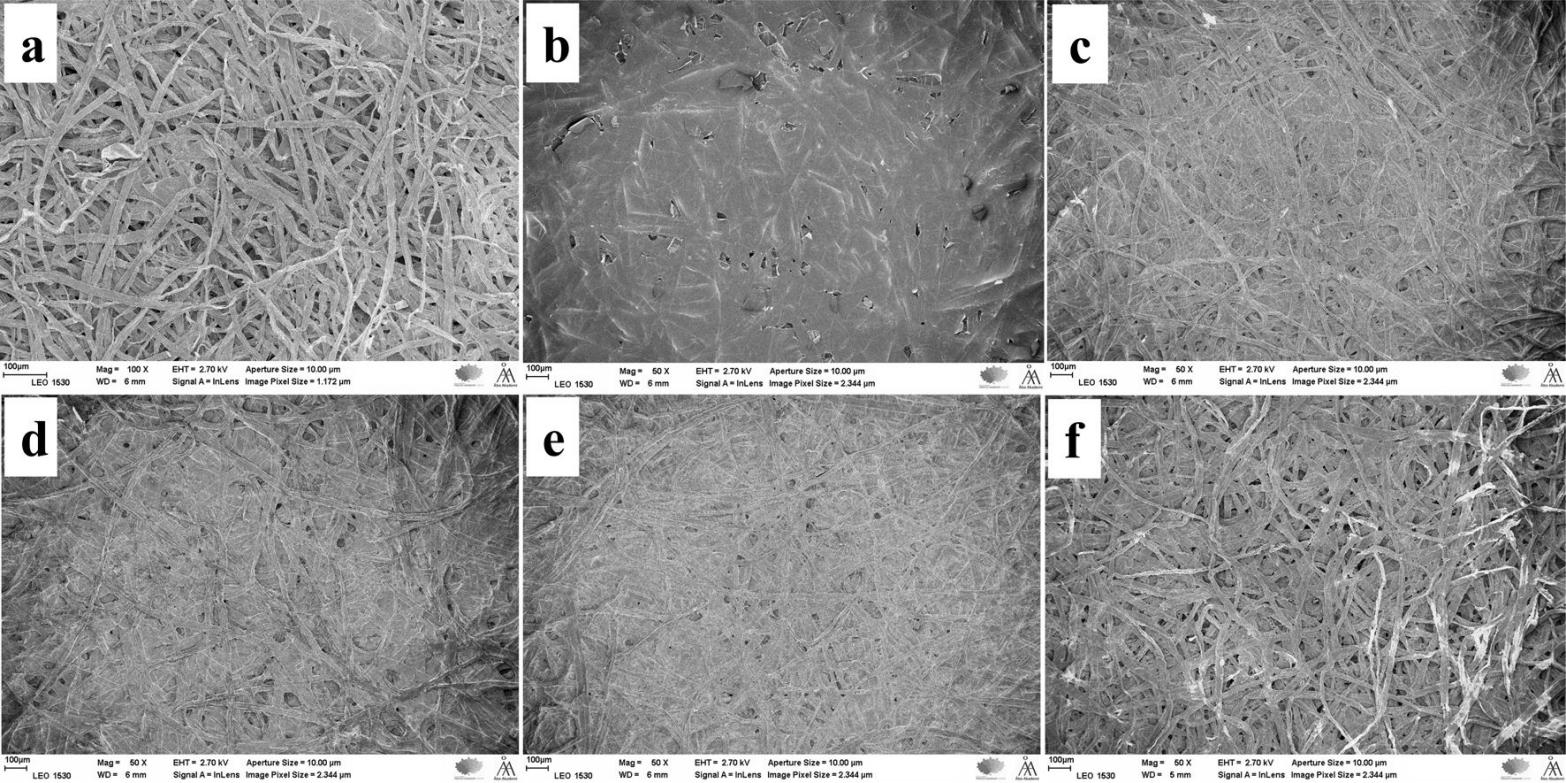
SEM images of the BKPR fibers: (a) REF (b) C-F (c) HF (d) C-HF (0.15 M) (e) C-HF (0.30 M) (f) C-HF (0.45 M).

**BKPR:** Upon refining, the reference fibers (REF) showed fibrils protruding out from the surface (Figure 6a). It is suggested that the mechanical force applied on the BKPR fibers leads to dissociative fibrillation. This treatment is expected to have increased the surface area of the fibers. The low-magnification images of unmodified fibers (REF, HF, Figure 6a,c) showed again a complex network of very fine cellulose fibers, which are smudged by the AA layer upon modification either directly or through LDH (C-F, C-HF) (Figure 6b and Figure 6d–f). The microscopy images suggest that the refinement has reduced the size of the fibers in length and thickness. The high-magnification images (Figure S2, Supporting Information File 1) of these samples show heavily wrinkled fiber surfaces for REF, HF (Figure S2b,d in Supporting Information File 1) compared to unrefined counterparts (Figure S1a,c in Supporting Information File 1), and a very thick, smooth, and shiny layer of AA on fibers directly modified with AA (C-F, Figure S2b, Supporting Information File 1). In contrast, C-HF they display a thin, transparent and shiny layer of AA coating (Figure S2d–f, Supporting Information File 1). Thus, the difference in morphology between refined and unrefined bleached kraft pulp fibers (BKP, BKPR) could play a role in inducing hydrophobicity and tensile strength.

**Figure 7:**
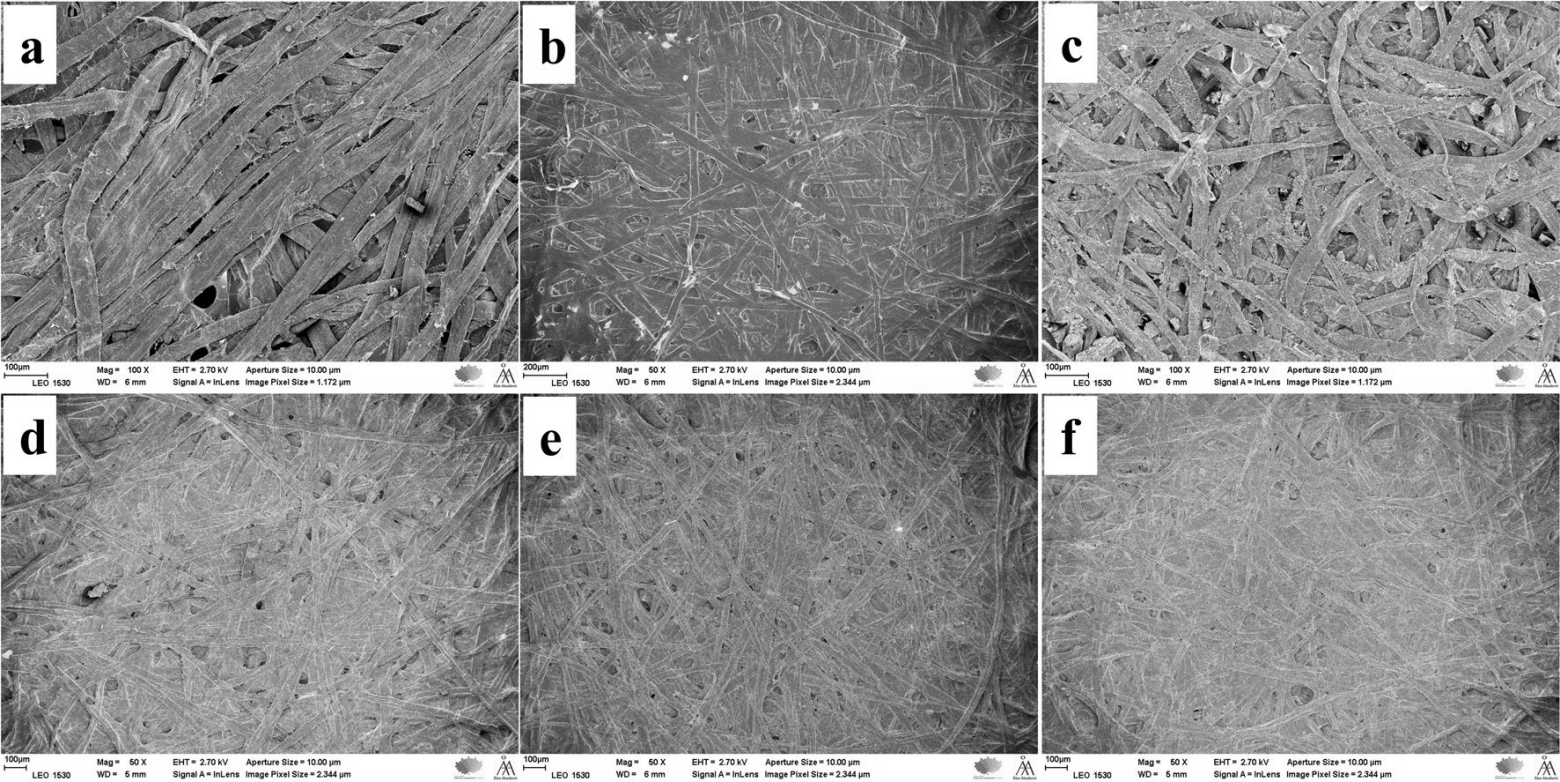
SEM images of the UBKP fibers: (a) REF (b) C-F (c) HF (d) C-HF (0.15 M) (e) C-HF (0.30 M) (f) C-HF (0.45 M).

**UBKP:** The reference fibers (REF) appear as tightly packed fiber bundles (Figure 7a). The HF show a web of thick fibers with LDH particles spread on the surface (Figure 7c). C-F fibers exhibit a thick covering of AA (Figure 7b). C-HF fibers reveal a moderately thick coating of AA (Figure 7d–f). The high-magnification images (Figure S3, Supporting Information File 1) reveal wrinkled surfaces or gel-like structures for all samples except C-F, which shows a very thick, smooth, and shiny coating of AA (Figure S3b, Supporting Information File 1). C-HF has a thick sticky glue-like coating of AA on the surface (Figure S3d–f, Supporting Information File 1).

**Figure 8:**
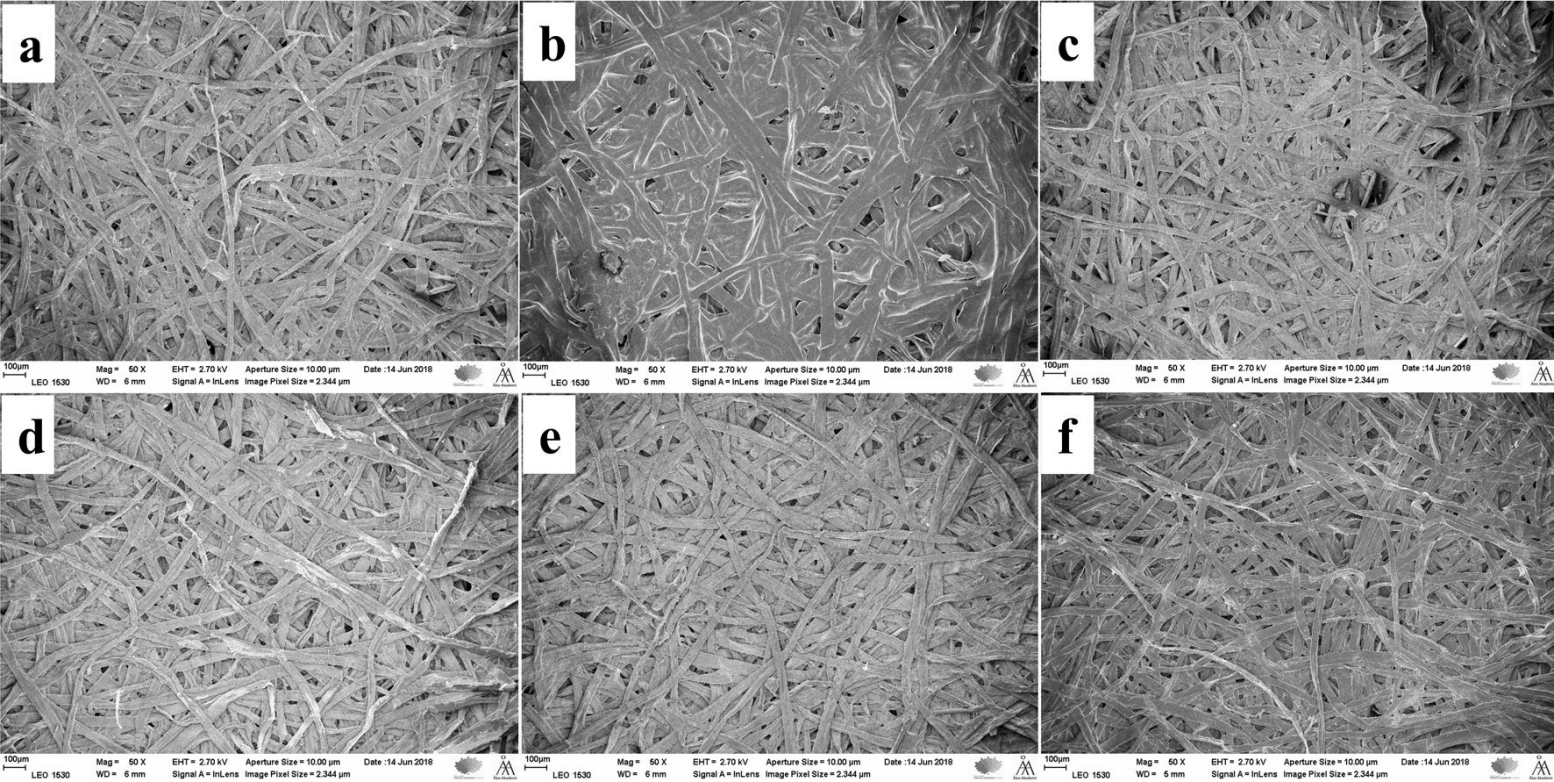
SEM images of the UBKPR fibers: (a) REF (b) C-F (c) HF (d) C-HF (0.15 M) (e) C-HF (0.30 M) (f) C-HF (0.45 M).

**UBKPR:** REF fibers show a dense complex web of longer and thicker cellulose fibers having hairy projections on the surface (Figure 8a). HF displays a similar appearance but with few LDH crystals present on the surface (Figure 8c). C-F reveal a very thick AA layer (Figure 8b), whereas C-HF fibers show only a minimal AA coating (Figure 8d–f). At higher magnification (Figure S4, Supporting Information File 1), the fibril projections of the refined fibers are clearly seen in all samples except C-F, which shows a very thick surface covering of AA wax (Figure S4b, Supporting Information File 1). C-HF fibers display a very thin covering of AA, compared to C-HF fibers from UBKP (Figure S4d–f, Supporting Information File 1).

The SEM characterization of materials suggests that unrefined fibers (BKP, UBKP) yield a better uptake of AA with uniform coating/adhesion than refined fibers (BKPR, UBKPR). SEM images of C-HF_0.30M_ fibers from BKP, BKPR, UBKP, UBKPR are compared in Figure S5 (Supporting Information File 1). All these samples exhibited a wax-like coverage of abietic acid on the fiber skeleton. The thickness of the AA coverage may not be the same in all materials, quantitatively. Depending on the thickness of the AA coverage and intrinsic hydrophobic content such as hemicellulose and lignin, these materials showed different performance in CA_water_ and oil absorption studies.

### Material Testing

#### Water contact angle (CA_water_)

**Figure 9:**
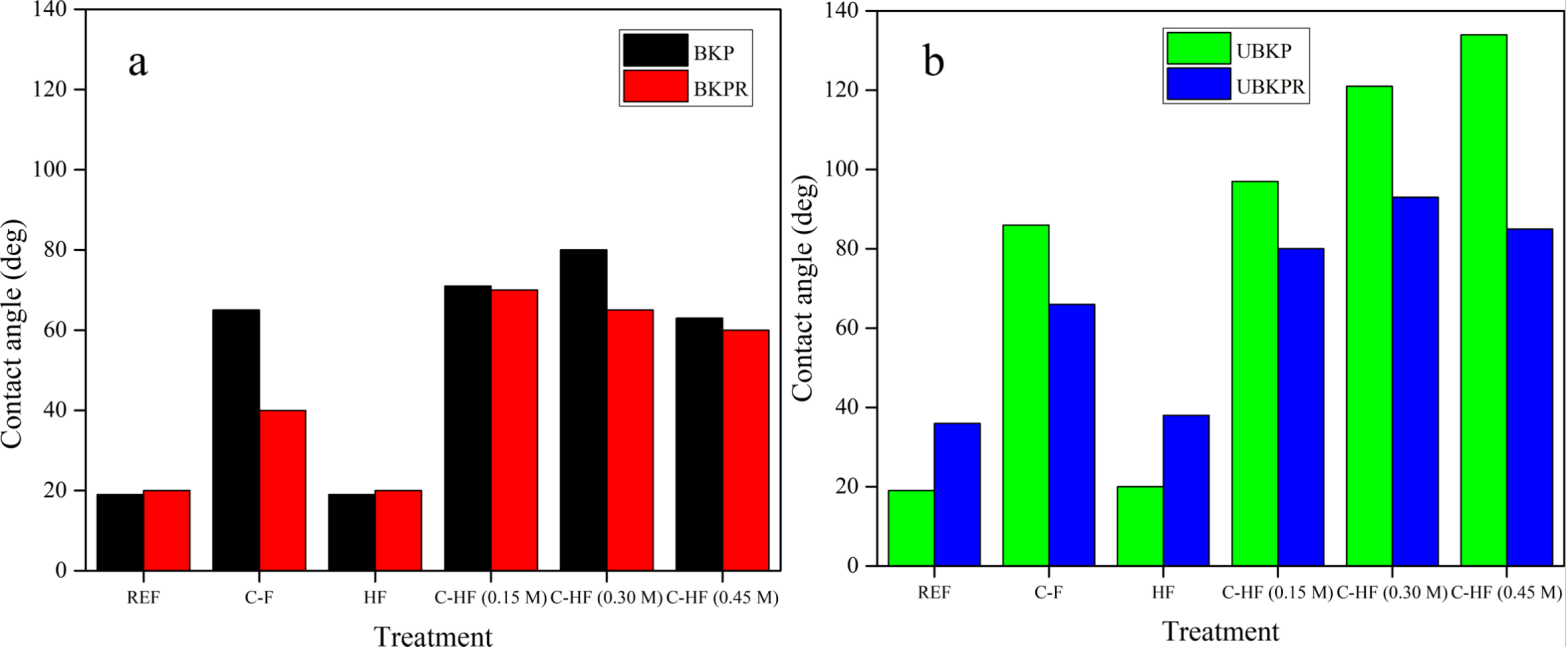
Water contact angle for (a) BKP and BKPR, (b) UBKP and UBKPR.

The fibers REF, C-F, HF, C-HF_0.15M_, C-HF_0.30M_, and C-HF_0.45M_ of all pulps were investigated with regard to their hydrophobicity in terms of water contact angle (Figure 9).

The REF fibers of BKPR, UBKPR exhibited higher CA_water_ values (20°, 36°) than the unrefined (BKP, UBKP) counterparts (19°, 19°, Figure 9). This suggests that the refining process opens up new fibrils on the surface of fibers, which increases the exposure of not only cellulosic –OH groups but also of hydrophobic lignin and hemicellulose. The latter increase the hydrophobicity of the fibers, and are removed during the bleaching process. Hence, the hydrophobicity is only associated with the intrinsic nature of cellulose.

Cellulose fibers directly modified with abietic acid (C-F) were also investigated. Refined C-F fibers (BKPR, UBKPR) showed a lower value of CA_water_ (40°, 66°) than the unrefined C-F fibers (BKP, UBKP) (65°, 86°). Refining caused a decrease in CA_water_ because of the deep diffusion and adsorption of AA inside the opened pores of the fibrillated surface. This leads to a smaller concentration of AA on the surface of the refined fibers. The same phenomenon occurs in bleached fibers. Hence, C-F fibers of UBKP were more hydrophobic (86°) than C-F fibers of BKP (65°). In contrast, unrefined unbleached fibers (UBKP) had a high concentration of AA on the surface and, hence, showed a higher value of CA_water_.

The LDH hybridized cellulose fibers (HF) showed similar trends as the reference fibers (REF), with refined LDH-containing fibers of bleached and unbleached pulps (BKPR, UBKPR) exhibiting higher hydrophobicity (20°, 38°) than the unrefined counterparts (BKP, UBKP, 19°, 20°) due to the increased exposure of hydrophobic lignin and hemicellulose (Figure 9). Refining increased the CA_water_ value by about 18° from bleached to unbleached fibers. The reason for the identical trend was that the LDH material was grafted on cellulose at the hydrophilic sites because of its self-assembly on cellulosic –OH groups via hydrogen bonding. Although the cellulosic –OH groups were masked by –OH groups at the bottom planes of LDH, the freely exposed –OH groups at the top planes of LDH maintain the intrinsic hydrophilic character of cellulose fibers. Hence, there was no much change in the balance of the intrinsic hydrophobic character of the pulp fibers.

**Figure 10:**
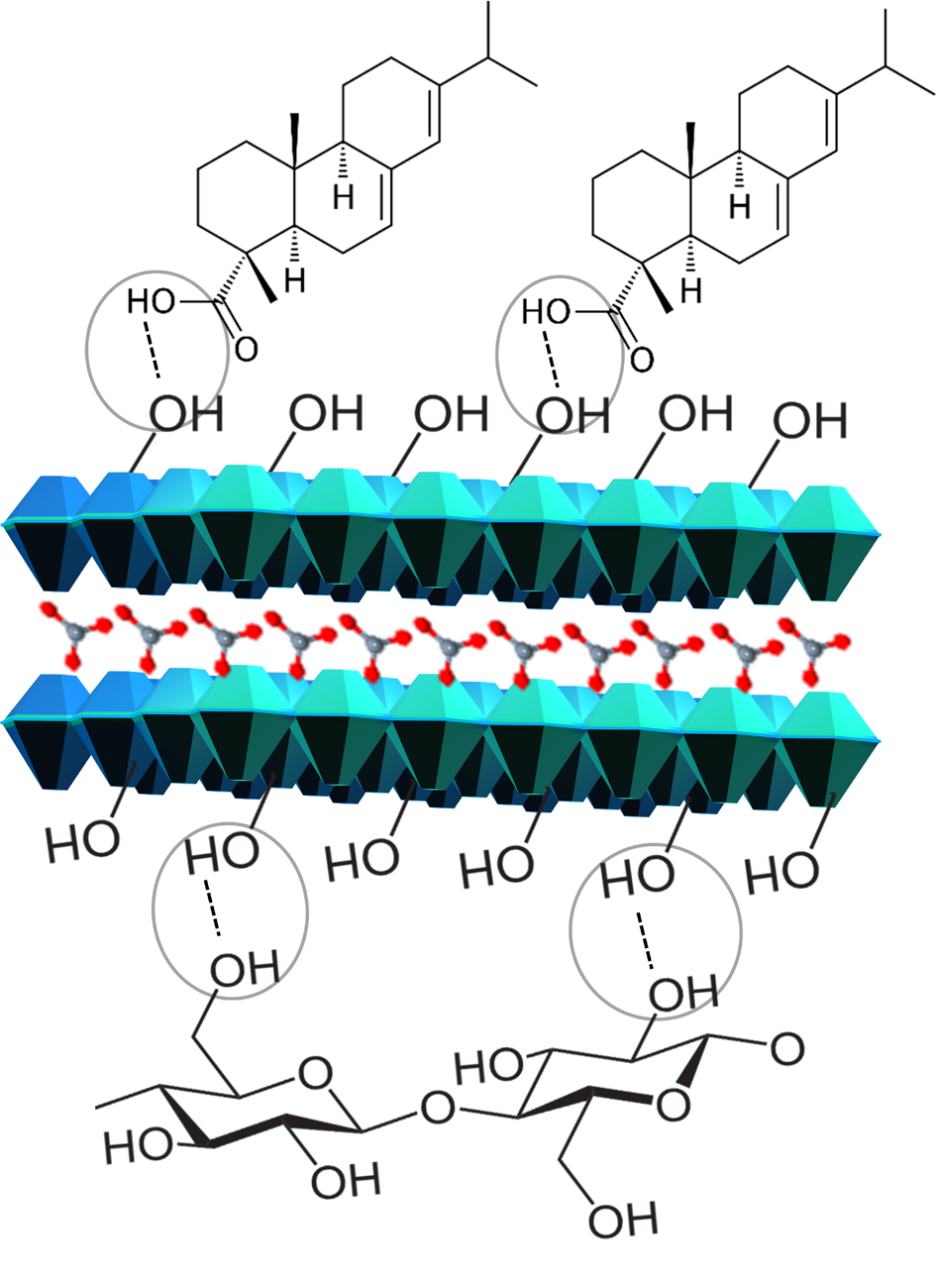
Hydrogen bonding directed assembly of AA on HF.

Composite hybrid fibers (C-HF) show higher water contact angle values than the two-component fibers, C-F and HF (Figure 9). The C-HF_0.15M_, C-HF_0.30M_, and C-HF_0.45M_ fibers of BKP, UBKP have higher values of water contact angle (71°, 97°) (80°, 121°) (63°, 134°) than the refined counterparts (BKPR, UBKPR) (70°, 80°) (65°, 93°) (60°, 85°). These results were attributed to the fact that AA moieties diffuse deep inside the opened pores of the refined fibers leading to lower hydrophobicity. The unrefined fibers (BKP, UBKP) have a high amount of AA on the surface increasing hydrophobicity. Again, unbleached fibers are not free from hemicellulose and lignin. These hydrophobic moieties add up to the performance of the materials in CA_water_ measurements. Regarding the structure of C-HF fibers, –COOH groups of AA have directed the AA molecules towards the top-plane –OH groups of LDH through hydrogen bonding (Figure 10). Hence, the AA modification on pulp fibers via the linker material LDH (C-HF) is intact and stronger than the direct AA modification on pulp fibers (C-F). In C-F, there could be always repulsion between cyclic carbons of cellulose and AA, which will also diminish the AA grafting on pulp fibers.

#### Oil absorption capacity

The oil absorption experiments were carried out with the idea that when the material is hydrophobic it should also be lipophilic. The results show that the water contact angle is proportional to the oil absorption (Table 3). Higher CA_water_ values lead to a higher oil absorption. C-HF fibers have stood out as best candidates for oil absorption. Among all, the C-HF fibers of BKP and UBKP exhibited superior performance of 189% and 295% oil absorption, respectively (Table 3). These hydrophobic cellulose materials can be utilized as water-proof materials or oil sorbents [14].

**Table 3:** Water contact angle and oil absorption values of the pulp fibers.

Pulp fibers	BKP		BKPR	
	CA_water_ (°)	Oil absorption (%)	CA_water_ (°)	Oil absorption (%)

REF	19	45	20	55
C-F	65	57	40	78
HF	19	55	20	63
C-HF (0.15 M)	71	154	70	147
C-HF (0.30 M)	80	189	65	97
C-HF (0.45 M)	63	145	60	92

Pulp fibers	UBKP		UBKPR	
	CA_water_ (°)	Oil absorption (%)	CA_water_ (°)	Oil absorption (%)

REF	19	78	36	50
C-F	86	109	66	67
HF	20	90	38	54
C-HF (0.15 M)	97	128	80	102
C-HF (0.30 M)	121	184	93	153
C-HF (0.45 M)	134	295	85	132

#### Tensile strength

**Figure 11:**
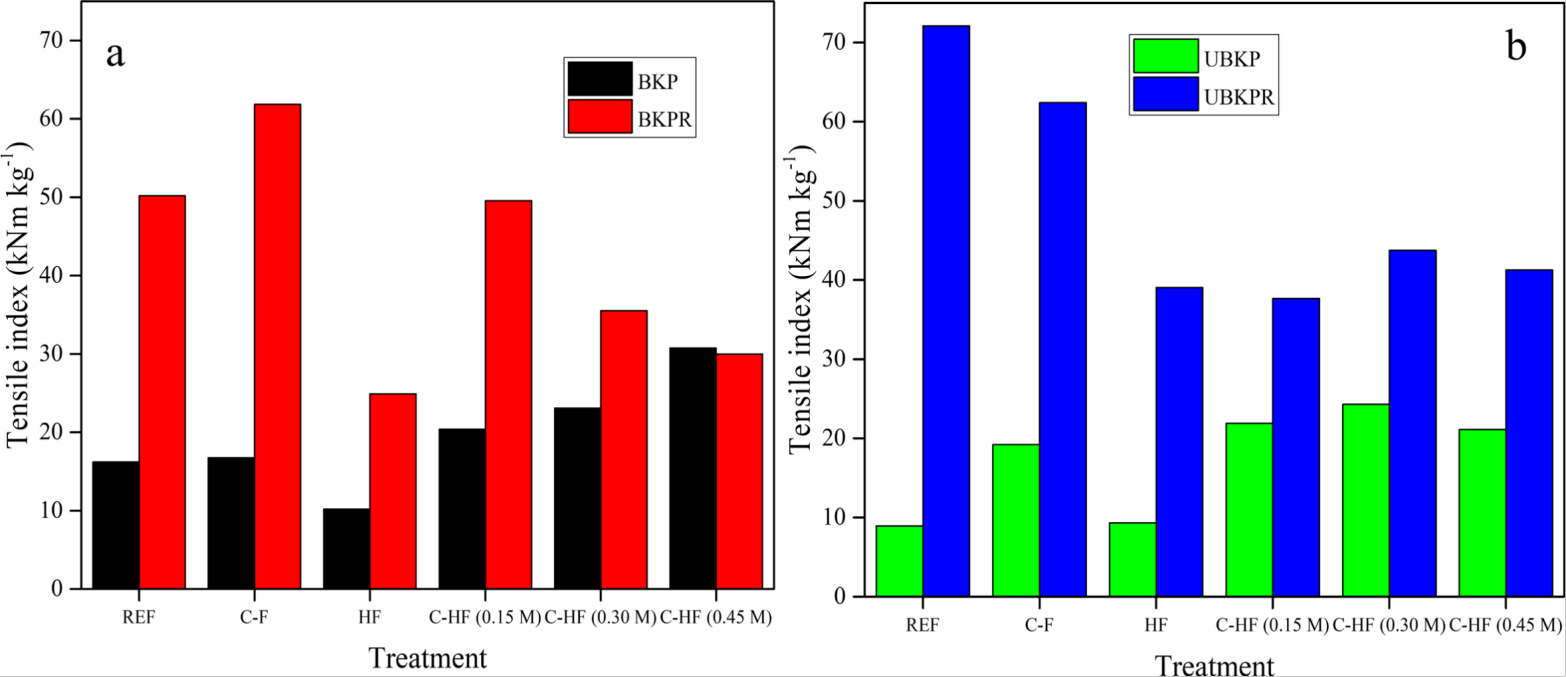
Tensile strength index for (a) BKP and BKP-R, (b) UBKP and UBKP-R.

The reference and modified fibers was also studied with regard to tensile strength (Figure 11). The refined REF fibers of bleached and unbleached pulp (BKPR, UBKPR) show higher values of the tensile strength index (tensile strength divided by grammage) (50.18 and 72.10 kNm·kg^−1^) than the unrefined counterparts (BKP, UBKP) (16.21 and 8.93 kNm·kg^−1^) (Table S1, Supporting Information File 1). This observation was directly opposite to the trend obtained in the water contact angle/oil absorption measurements, where the unrefined fibers showed high hydrophobicity. However, it was highly reasonable that the refining process, which shortens the length of the fibers and opens up new fibrils on the surface, makes the fiber network very complex and tight binding. Moreover, the increased hydrogen bonding (–CH*_n_*–OH^…^OH–CH*_n_*–) between newly exposed –OH groups in refined fibers caused improved tensile strength. Further, the higher tensile strength index of the refined fibers, compared to the unrefined fibers, was maintained even after the treatments, i.e., in C-F, HF, and C-HF.

Regarding a possible increased tensile strength, the treatments (LDH hybridization and AA modification) have positive effects only on unrefined fibers of bleached and unbleached pulp (C-HF of BKP, UBKP). The interaction with refined fibers of bleached and unbleached pulp (C-HF of BKPR, UBKPR) either maintains or reduces the existing tensile strength (Table S1, Supporting Information File 1). Hence, the refined fibers do not need any chemical treatment to increase the tensile strength, while the unrefined fibers need chemical functionalization.

In the case of fibers directly modified with AA (C-F), the AA treatment improved the tensile strength only for BKPR, UBKP but not for BKP, UBKPR pulps (Table S1, Supporting Information File 1). This suggests that AA forms a constructive composite only under the condition of low amounts of lignin or hemicellulose. However, the improved tensile strength was obtained only with BKPR and not with UBKP pulp. This revealed that AA not only needed low amounts of lignin or hemicellulose but also a high surface area. Although UBKPR has a high surface area, it has also increased amounts of lignin of hemicelluloses. Hence, AA has a negative effect on it. The more lignin or hemicelluloses are present, the higher the repulsion with AA, due to the aromatic carbon rings. Hence, the AA coverage on these fibers is smaller, which was evident from SEM as well. Therefore, there is less AA on the fiber surface to bind the fibers for improved tensile strength.

The sole LDH treatment of fibers and subsequent sheet forming (HF) showed a decrease in tensile strength indices for most of the pulps (Table S1, Supporting Information File 1) compared to the respective unmodified reference fibers (REF). The LDH particles are synthesized in the presence of disintegrated individual fibers, leading to a homogenous distribution of LDH particles on the surface of the fibers. These LDH particles completely cover the underlying fiber (polysaccharide material), which usually tend to form strong hydrogen bonds with other fiber strands. This direct hydrogen bonding from fiber to fiber (–CH*_n_*–OH^…^OH–CH*_n_*–) is different and stronger than hydrogen bonding between LDH –OH groups and cellulosic –OH groups (M*^n^*^+^–OH^…^OH–CH*_n_*–) or –COOH groups of AA (M*^n^*^+^–OH^…^OHOC–CH*_n_*–), which plays a role in stacking AA and cellulose fibers above and below LDH, respectively. Thus, LDH hinders the inter-fiber bonding and reduces the tensile strength. However, this weakening of inter-fiber bonding was overcome by modifying hybrid fibers with AA. AA was self-assembled on LDH binding sites via hydrogen bonding (M*^n^*^+^–OH^…^OHOC–CH*_n_*–). Then the adhesive action of AA on the fiber surface increased the binding between separate strands of fibers and improved the tensile strength. However, this type of action proved to be advantageous only with unrefined fibers. With refined fibers, the refining action itself was sufficient to increase the tensile strength. The results show that refined pulp fibers (REF of BKPR, UBKPR) and C-HF prepared from unrefined pulps (BKP and UBKP) can be utilized in reinforcement applications.

#### ISO brightness

**Figure 12:**
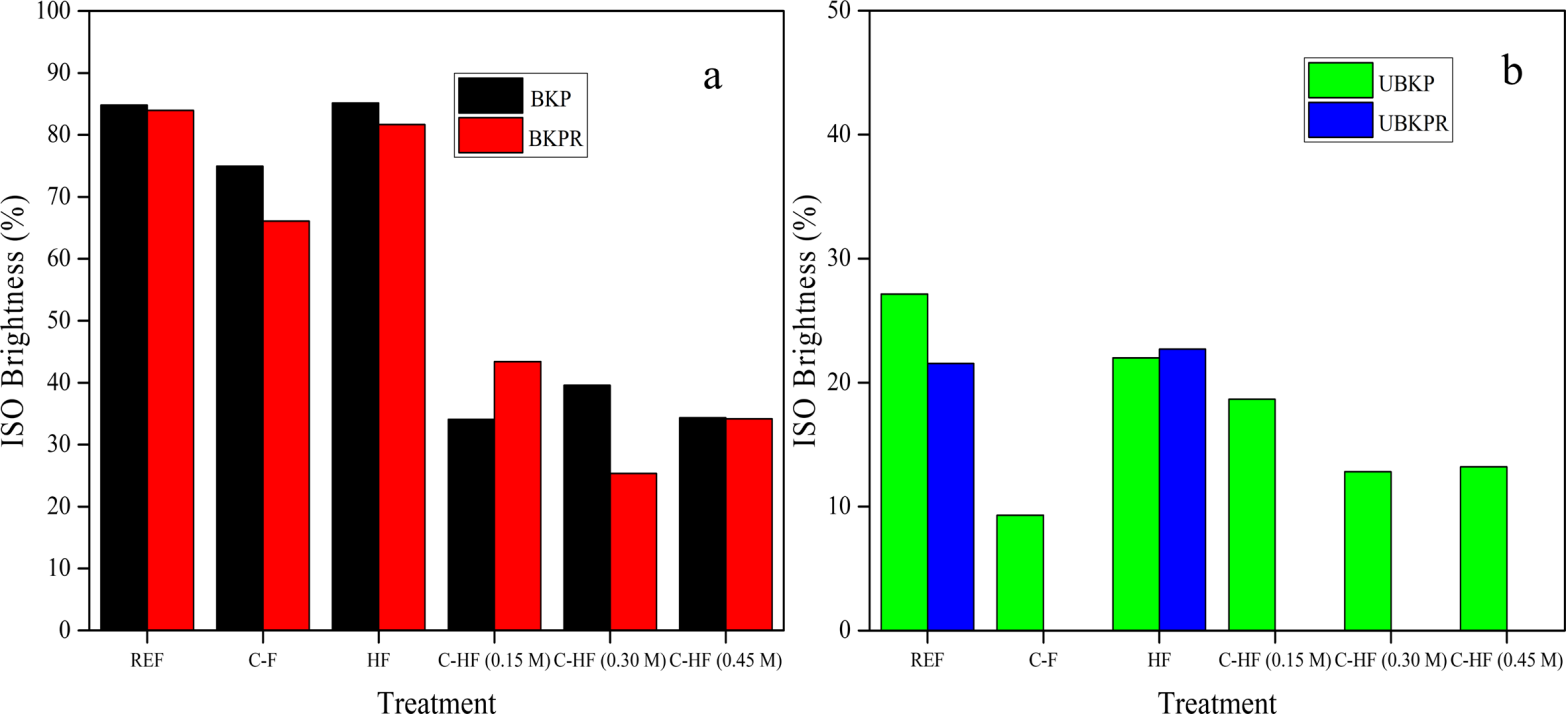
ISO Brightness of (a) BKP and BKP-R and (b) UBKP and UBKP-R.

As expected, the ISO brightness was found to be higher with bleached pulp compared to unbleached pulp (Figure 12). Bleaching essentially removes lignin and hemicelluloses that absorb light and cause the dark appearance of pulp. The refined fibers of bleached and unbleached pulp (BKPR, UBKPR) exhibited slightly less brightness (83.96%, 21.54%) than the unrefined counterparts (BKP, UBKP) (84.80%, 27.15%) (Table S2, Supporting Information File 1). This may be because the refined fibers have more empty spaces and pores than unrefined fibers, which might reduce light scattering and reflectance. The brightness of unbleached pulps (UBKP, UBKPR) was markedly lower. UBKPR shows a very poor ISO brightness which sometimes could not be determined due the very weak reflectance (Table S2, Supporting Information File 1).

C-F fibers exhibit a lower brightness than the reference fibers (REF, Table S2, Supporting Information File 1). Again, the difference was huge with unbleached fibers for the reasons discussed above. AA, which is a resin acid with dull brown color, absorbed light even more. The hybrid fibers (HF) showed almost identical brightness values compared to reference fibers (REF) because LDH (white in color) reflects light. The combination of AA and LDH (C-HF) reduced the ISO brightness values (Table S2, Supporting Information File 1).

#### Comparing the data with our previous report

The use of abietic acid has significantly increased CA_water_ values and oil absorption, which we aimed for. In the experiments with stearic acid (SA) in our previous work, the composite material reach a maximum CA_water_ value of 150° after 24 h of treatment. In contrast, with abietic acid (AA) the HF material reach a CA_water_ value of 134° after only 15 min of treatment. The corresponding oil absorption percentage weight increases were 155% and 295%, respectively. This means AA-modified HF absorbs adsorb twice the amount of oil compared to SA-modified HF fibers. Another important reason to pick abietic acid was to improve the tensile strength, as the SA-modified HF lost 84% of its tensile strength compared to the reference fibers (REF). The tensile strength percentages after modification with AA are 90% increase, 1% decrease, 172% increase and 39% decrease for BKP, BKPR, UBKP and UBKPR, respectively. Thus, abietic acid has a better performance with regard to tensile strength than stearic acid.

## Conclusion

The abietic acid (AA)-induced hydrophobicity on bleached pine kraft pulp and unbleached spruce kraft pulp fibers (F), refined and unrefined, requires a linker material, i.e., a layered double hydroxide (LDH). LDH, self-assembles on cellulose fiber surface through hydrogen bonding on the bottom plane and further directs AA to graft on it via hydrogen bonding but this time on the top plane. Thus, a sandwich composite hybrid material (C-HF) is formed. C-HF materials exhibit a better performance in water-contact angle (CA_water_) and oil absorption measurements than unmodified/reference fibers, fibers modified with only AA (C-F) or only LDH (HF). C-HF obtained from unrefined fibers showed higher CA_water_ values than refined fibers, as the refined fibers have a reduced surface concentration of AA due to diffusion of AA inside the pores. In contrast, refined fibers exhibit higher tensile strength than unrefined fibers, due to the newly opened fibrils and surface –OH groups available for hydrogen bonding/inter-fiber binding. The refining is sufficient to achieve improved tensile strength in pulp fibers. Thus, treatment with LDH and AA is required only for improving the tensile strength of unrefined fibers. As expected, the ISO brightness of bleached fibers is higher than that of unbleached fibers due to the smaller content of lignin and hemicellulose. In addition, the unrefined fibers are brighter than refined fibers, as there are less empty space of pores that reduce the reflectance. The present investigation of pulp fibers can be taken to the next level at which the improved hydrophobicity and tensile strength could be utilized for applications in the field of sorption or reinforcement.

## Supporting Information

Supporting Information contains additional SEM figures, and tensile strength and ISO brightness data that are relevant to the discussion part.

File 1 Additional experimental data.
