# Supplementary material for: Topochemical engineering of composite hybrid fibers using layered double hydroxides and abietic acid
Source: Beilstein J Nanotechnol. 2019 Feb 28;10:589–605. doi: 10.3762/bjnano.10.60 (PMC6404409; doi:10.3762/bjnano.10.60)
Supplement: File 1 — Additional experimental data. [file Beilstein_J_Nanotechnol-10-589-s001.pdf]

BEILSTEIN JOURNAL OF NANOTECHNOLOGY

Supporting Information

for

Topochemical engineering of composite hybrid fibers using layered double hydroxides and abietic acid

Liji Sobhana, Lokesh Kesavan, Jan Gustafsson and Pedro Fardim

Beilstein J. Nanotechnol. **2019,** *10,* 589–605. doi:10.3762/bjnano.10.60

Additional experimental data

License and Terms: This is a supporting information file under the terms of the Creative Commons Attribution License (http://creativecommons.org/licenses/by/4.0). Please note that the reuse, redistribution and reproduction in particular requires that the authors and source are credited.
The license is subject to the *Beilstein Journal of Nanotechnology* terms and conditions: (https://www.beilstein-journals.org/bjnano)

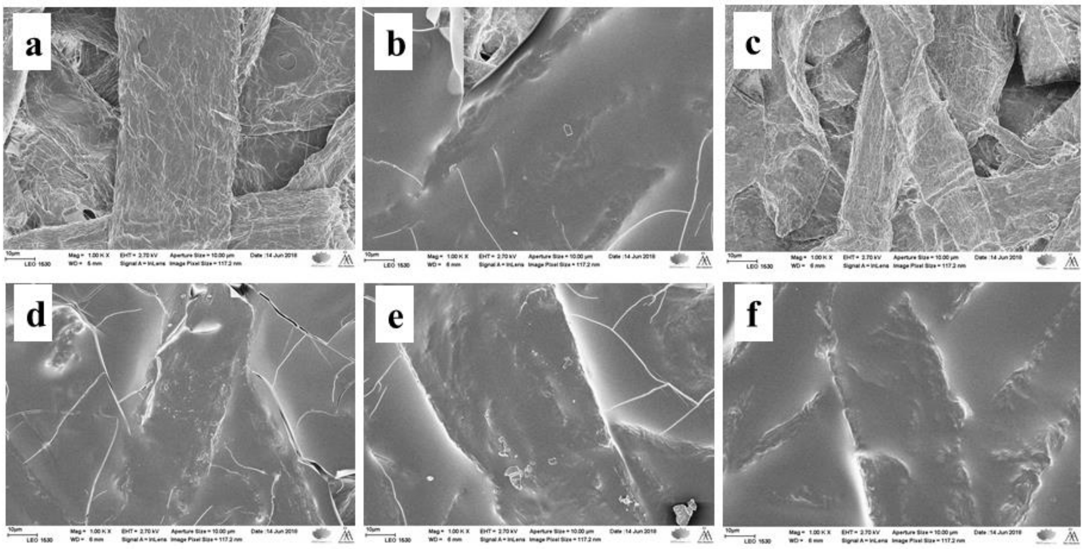

Figure S1: SEM images of the BKP fibres (a) REF (b) C-F (c) HF (d) C-HF (0.15 M) (e) C-HF (0.30 M) (f) C-HF (0.45 M).

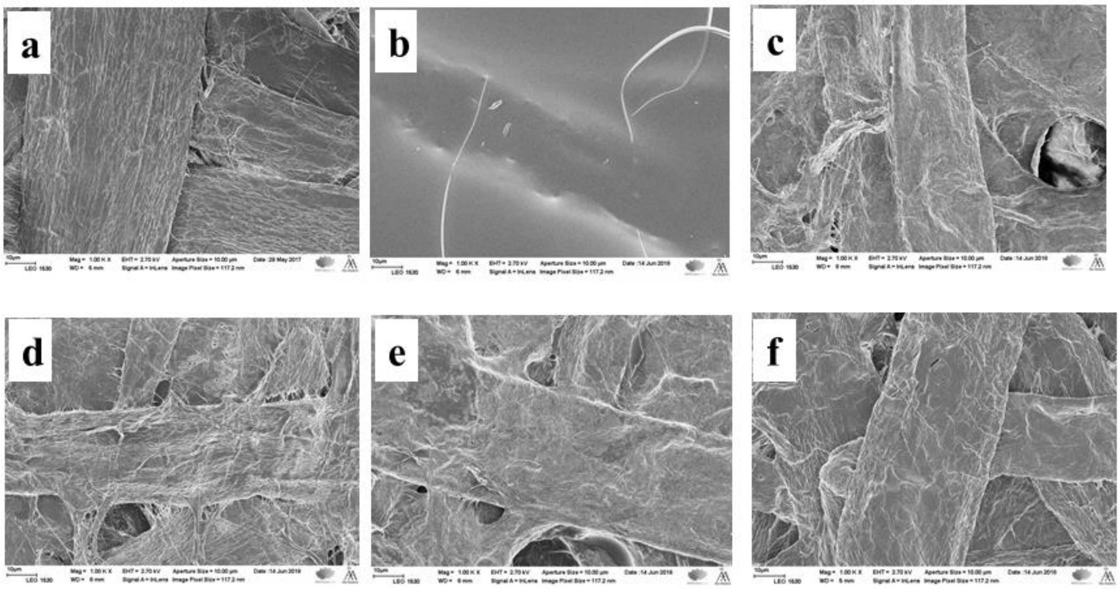

Figure S2: SEM images of the BKPR fibres (a) REF (b) C-F (c) HF (d) C-HF (0.15 M) (e) C-HF (0.30 M) (f) C-HF (0.45 M).

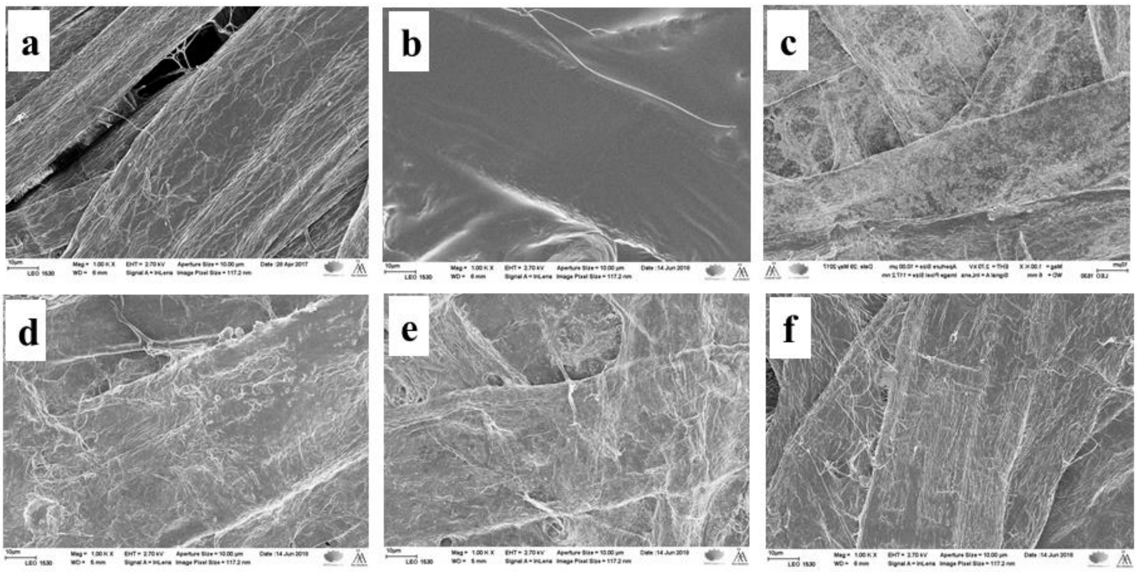

Figure S3: SEM images of the UBKP fibres (a) REF (b) C-F (c) HF (d) C-HF (0.15 M) (e) C-HF (0.30 M) (f) C-HF (0.45 M).

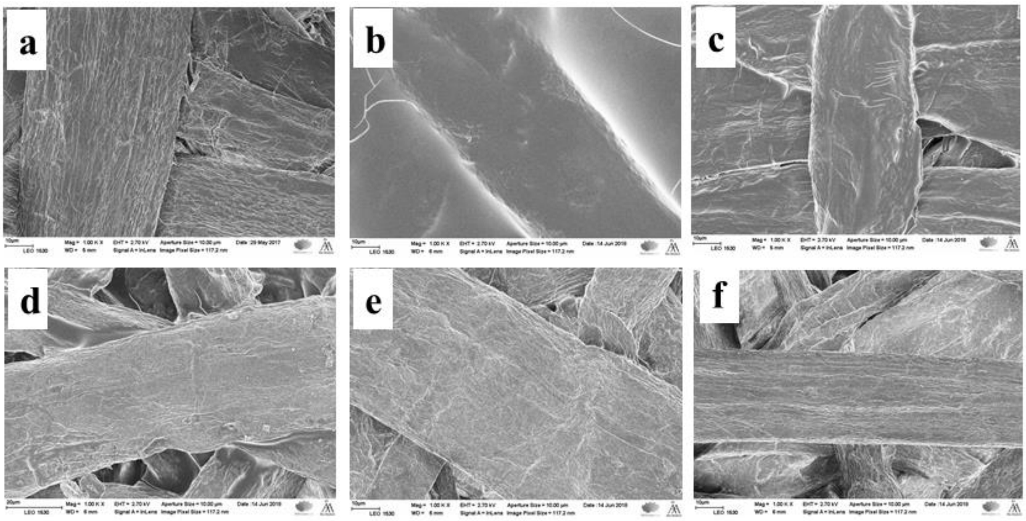

Figure S4: SEM images of the UBKPR fibres (a) REF (b) C-F (c) HF (d) C-HF (0.15 M) (e) C-HF (0.30 M) (f) C-HF (0.45 M).

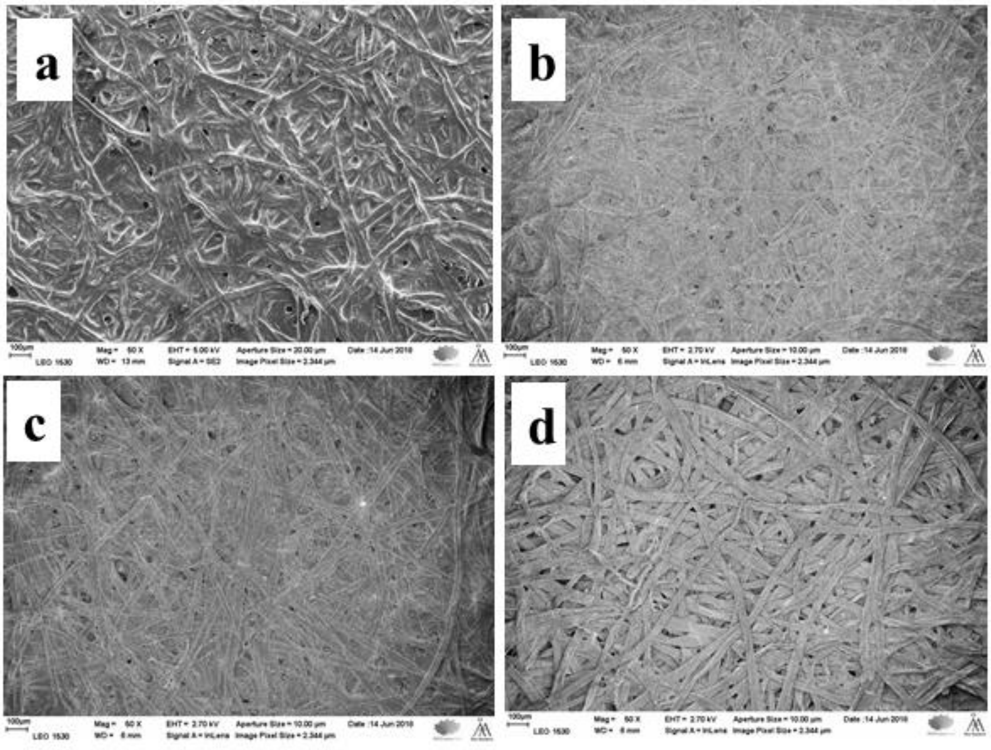

Figure S5: SEM images of C-HF under same grafting conditions (a) BKP (b) BKPR (c) UBKP (d) UBKPR.

Table S1: Tensile strength index of pulp fibers.

	Tensile strength index (kNm kg^{-1})			
**Material**	**BKP**	**BKPR**	**UBKP**	**UBKPR**
REF	16.21	50.18	8.93	72.10
C-F	16.73	61.84	19.22	62.41
HF	10.21	24.91	9.306	39.04
C-HF (0.15M)	20.40	**49.57**	21.88	37.68
C-HF (0.30M)	23.09	35.53	**24.28**	**43.74**
C-HF (0.45M)	**30.75**	30.00	21.11	41.26

Table S2: ISO brightness of pulp fibers.

	ISO brightness (%)			
**Material**	**BKP**	**BKPR**	**UBKP**	**UBKPR**
REF	84.80	83.96	27.15	21.54
C-F	74.95	66.09	9.31	ND
HF	85.16	81.67	21.99	22.70
C-HF (0.15M)	34.09	43.40	18.65	ND
C-HF (0.30M)	39.58	25.34	12.81	ND
C-HF (0.45M)	34.34	34.17	13.21	ND
